# The Role of Systemic Family Psychotherapy in Glycemic Control for Children with Type 1 Diabetes

**DOI:** 10.3390/children11010104

**Published:** 2024-01-15

**Authors:** Andreea Salcudean, Maria Melania Lica

**Affiliations:** Department of Bioethics, Social and Human Sciences, University of Medicine and Pharmacy, Science and Technology George Emil Palade of Targu Mures, Gheorghe Marinescu Street No. 38, 540142 Targu Mures, Romania; andreea.salcudean@umfst.ro

**Keywords:** type 1 diabetes mellitus, glycemic control, disease management, family systemic psychotherapy

## Abstract

(1) Background: Family factors play an important role in the management of diabetes, establishing a relationship between conflicts and non-adherence to therapy. High values of HbA1c are involved in specific complications of the disease (retinopathy, nephropathy, neuropathy, ketoacidosis). This study aimed to determine the role of systemic family psychotherapeutic interventions in increasing the quality of parent–child/adolescent relationships and in optimizing the child’s glycemic control. (2) Methods: In this prospective observational study, 64 parents of children and adolescents with type 1 diabetes were evaluated regarding their relationship with their children, using the Child–Parent Relationship Scale-Short Form (CPRS-short form). The children were divided into three groups: one participated for 6 months in systemic family psychotherapy with children and their parents (FT), the second group participated in individual psychotherapy (IT), and the control group (CG) received no intervention. HbA1c values were recorded before and after the interventions. (3) Results: HbA1c means decreased significantly after the family psychotherapy program. The scores on closeness in the family therapy group increased significantly, and the scores on conflict decreased significantly after the intervention, compared with IT and CG. (4) Conclusions: Systemic family psychotherapy produces better results in disease management and in strengthening parent–child relationships.

## 1. Introduction

Diabetes is a chronic disease characterized by the inability of the pancreas to produce the necessary insulin because beta cells are damaged or insufficiently functioning [1]. The direct effect is a high blood glucose level that negatively affects the micro- and macrovascular systems. It was estimated that, in 2021, 1.2 million children and adolescents worldwide had diabetes. In Europe in the same year, there were 295,000 registered cases of T1DM. A total of 3860 cases were diagnosed in Romanian children and adolescents [2].

In type 1 diabetes mellitus (T1DM), treatment protocol and disease management aim to monitor blood glucose, insulin administration via multiple injections or insulin pumps, diet, and physical activity [1].

The glycosylated hemoglobin (HbA1c) value is a reliable tool for the diagnosis of diabetes and the evaluation and periodic monitoring of patients’ glycemic control [2]. It represents the average blood glucose level for 1–3 months prior to collection. To ensure the validity of the results, the test devices must be approved by the National Glycohemoglobin Standardization Program [2]. High values of HbA1c indicate high blood glucose levels occurring several times in the observed time period, low metabolic control, and difficulties in managing the disease; these values predict the risk of complications specific to the disease (retinopathy, nephropathy, neuropathy, ketoacidosis) [2,3]. The optimal value of HbA1c in patients with T1DM is 6.5–7%, and the risk value in the undiagnosed population is 5.7–6.4 [3]. Another means of determining the degree of glycemic management is continued glycemic monitoring (CGM). This is considered more valuable because several blood glucose measurements are taken over 24 h, and the upward or downward trend of blood sugar levels is tracked [4]. The disadvantage of this method is that it is not equally accessible to all patients [5]. Therapeutic education concentrates on increasing the awareness of the importance of a stable glycemic balance in patients and their caregivers [5]. The difficulty in achieving these goals creates feelings of ineffectiveness, powerlessness, helplessness, and frustration in both children and parents [6,7]. This association of negative thoughts and emotions contributes to the development or accentuation of negative affective states, and family conflicts and failures in social relationships or in personal development [8].

Although the biomedical approach is extremely important in the management of type 1 diabetes mellitus, it cannot singularly account for the complexity of interindividual differences in adherence to therapy and disease management [9]. Only an integrative approach to the disease, focusing on the interactions among biological, physiological, psychological, behavioral, environmental, and sociocultural factors, can build a complete picture of the clinical case and lead to personalized therapy [10].

Research has shown that family dynamics play an important role in the management of diabetes in children and adolescents, establishing a relationship between conflicts and non-adherence to therapy [11]. A chronic illness, infectious disease, mental illness, or special conditions such as pregnancy or separation may cause changes that, most of the time, disrupt the normal functioning of the families. They are then forced to reorganize their lifestyle, schedule, priorities, habits, roles, and other characteristics to face the associated daily challenges and complex psychological difficulties [12,13,14].

Patients with chronic diseases, and the people who take care of them, may suffer from mood disorders that negatively influence their involvement in treatment and the ability to communicate assertively with others [15,16]. Often, these families tend to avoid or limit social interactions due to the limited free time and responsibilities dictated by the treatment routine. The consequences are social isolation and a reduced social support network [17,18,19]. Regarding the stress perceived by parents concerning their son’s/daughter’s disease, a study revealed that 47% of parents believed that their professional activity and career had been heavily affected after their child was diagnosed with T1DM, and 33% stated that diabetes had had a negative impact on the financial balance of the family [19].

The conceptualization of the disease and the self-efficacy perceived in managing it can generate feelings of guilt, anger, anxiety, helplessness, depression, or even exhaustion, especially in caregivers, who are most often the child’s mother [20,21]. These irrational thoughts can generate hyper-protective behavior with an exclusive focus on disease management, which ignores the child’s or the parent’s own psychological needs [22,23].

Perfectionism and excessive parental criticism have been associated with low self-esteem and low self-care interest in preadolescents [24]. Controversies regarding the effects of parental criticism on glycemic control were highlighted by other studies, which considered a critical attitude less important and the emotional involvement of parents more significant [20,25,26].

In another study of the parents of children under 7 years, parents’ perceived stress and the quality of their relationship with their children were more associated with their quality of life and less with their HbA1c value, but it was not possible to explain this correlation [27]. Much of the literature on the conflict between parents and children or adolescents with T1DM focuses on assessing divergence regarding diabetes, and less on improving the quality of their relationship, strengthening it, collaborating, and increasing parental support and confidence as a basis for successfully overcoming current or future critical situations [24,25,26,27].

In medical practice, psychological interventions in T1DM for children and adolescents, and their families aim to increase adherence to treatment and decrease the risk of disease-specific complications, as well as to identify internal and external protective factors that affect the achievement of these goals and increases their quality of life [1,2,8]. In Romania, this necessity is rarely considered, and only individual intervention is used.

The aim of this study was to determine the role of systemic family psychotherapeutic interventions in increasing the quality of relationships of parent (caregiver)–child/adolescent with T1DM from Romanian families, and optimizing their glycemic control.

The objectives were:

- To build and offer a psychotherapeutic intervention program based on systemic psychotherapy to the families of children and adolescents with T1DM, to improve the children’s relationship with their parents and increase glycemic control;
- To identify the perceived changes in the quality of the parent’s relationship with children and adolescents with T1DM, utilizing family systemic psychotherapeutic intervention programs and individual psychotherapeutic interventions, compared to a control group;
- To assess differences in glycemic control, utilizing family systemic psychotherapeutic intervention programs and individual psychotherapeutic interventions, compared to a control group.

Hypotheses:

**H1.**  *The quality of the relationship between the parent and child/adolescent with T1DM increases significantly after their co-participation in systemic psychotherapy sessions compared to the group in which only the child/adolescent participates in individual therapy, and to the group without intervention.*

**H2.**  *The HbA1c value of children and adolescents decreases significantly following a systemic family psychotherapy program with their parents (caregivers), compared to the group in which only the child/adolescent participates in individual therapy and to the group without intervention.*

## 2. Materials and Methods

### 2.1. Study Type and Participants

In this prospective observational study carried out between April 2022 and October 2022, 110 children and adolescents with T1DM from Mures County, Romania, aged from 7 to 18 years old, were initially recruited. Among them, 46 children were excluded due to not meeting the participation criteria, leaving 64 children in the study. Additionally, included in the study for each participating child and adolescent was the parent who cared for them (caregiver) (N = 64). Informed consent and agreement to participate were obtained from all study participants, and all participants’ identity data were coded.

The criteria for the inclusion of children and adolescents in the study were: the confirmed diagnosis of T1DM for at least 10 months, the initial glycosylated hemoglobin (HbA1c) value higher than 7 mg/dL, the absence of other medical and/or psychiatric conditions, the ability to understand and communicate in Romanian, and the condition of not having previously participated in psychotherapy or psychological counseling sessions. The exclusion criteria for both parents and children were the following: refusal to participate in the study, absence from psychotherapy sessions, psychiatric history or psychological disorders (eating disorders, depression, anxiety, behavioral problems), previous participation in psychotherapy or psychological counseling, inability to write or understand Romanian, initial HbA1c analysis unavailable or inconsistent (older than 30 days), or final HbA1c analysis absent.

In the first part of the study (initial), all parents (N = 64):

1. Completed a demographic data questionnaire created by the authors of the study regarding the child’s age, gender, duration of the disease, environment (urban/rural); and the parent’s age, gender, marital status, education level, and family income;

2. Provided the latest glycosylated hemoglobin (HbA1c) value from the child’s personal medical record (not older than 30 days);

3. Participated in a semi-structured interview based on the questions in the CPRS Scale-SF (Child–Parent Relationship Scale-Short Form) created by Robert C. Pianta (1992), which facilitates the analysis of two opposing directions of the parent–child relationship: proximity and conflict [28]. This combined assessment option was preferred to a scenario where only the parents completed the answers, in order to collect additional information.

The interviews and therapy sessions were conducted face to face by the same specialist, a PhD psychologist, trained for 4 years in family systemic therapy, with 15 years of professional experience. The specialist evaluated the quality of the parent–child and parent–adolescent relationships; the questions having been adapted to these two age stages. The questions focused on the dimension of closeness (attachment, psychological comfort, physical affection, valorization, encouragement, openness, trust, sense of security, appreciation, communication) and conflict (arguments, ambivalent attachment, resistance, exhaustion, desire to “escape”, stubbornness, helplessness, manipulation, frequent mood swings) [28]. The parents’ answers were rated on a Likert scale from 1 to 5, where 1 “does not apply to us” and 5 “definitely apply to us”. During the interview, other relevant information was also recorded, including behavioral and emotional manifestations, insistences, avoidances, tone of voice (e.g., optimistic, tender, authoritative), positioning in the room, etc.

Each item of the Child–Parent Relationship Scale (according to Robert C. Pianta) applies to one of the two dimensions: closeness and conflict [28].

CLOSENESS: Q1—warm, affectionate relationship with the child; Q3—the child seeks the comfort of the parent when they are angry; Q5—the child values the relationship with the parent; Q6—the child’s pride when praised by the parent; Q7—the child shares information about his or herself with the parent; Q9—the ease of being in the same mood as the child; Q15—the child’s openness in sharing their own emotions and experiences with the parent.

CONFLICT: Q2—the parent and the child seem to be in a continuous struggle; Q4—the child’s discomfort with the parent’s physical condition; Q8—the child easily gets angry with the parent; Q10—the child remains angry/resistant after being disciplined; Q11—the parent feels exhausted in communicating with the child; Q12—the persistence of the child’s negative mood throughout the day; Q13—the child’s feelings toward the parent are unpredictable or changeable; Q14—manipulation of the parent by the child.

The answers were recorded during the interviews, with the consent of the participants, and partially coded. Later, these were deleted.

In the intervention phase, the 64 children and adolescents with T1DM were randomly divided into three groups: Group 1 (FT) (N = 22), which benefited from a total of 12 bimonthly sessions of systemic family psychotherapy together with the accompanying parent (90 min/session); Group 2 (IT) (N = 22), in which only children and adolescents participated in 12 bimonthly individual psychotherapy sessions (50 min/session); and the control group (CG), which did not participate in any psychotherapeutic intervention (N = 20). Thirty days after the end of psychotherapeutic interventions (family and individual), all parents participated in a new semi-structured interview, based on the same questions, and a new HbA1c value was recorded for each child and adolescent participating in the study.

### 2.2. Measurement of Glycated Hemoglobin

The glycated hemoglobin was analyzed from an integral blood sample drawn by venipuncture. Blood processing was performed in a certified clinical laboratory (Bioclinica Laboratories, Târgu Mures, Romania). The method involved Ion-exchange HPLC/Turbidimetry/Liquid chromatography high performance/Immunoturbidimetry, according to the manufacturer’s instructions.

### 2.3. Intervention

The sessions of systemic family psychotherapy took place every 2 weeks for 6 months (12 sessions/90 min each). The approaches used to organize the intervention program were psychodynamic, multigenerational, communicational, experiential, strategic, structural, behavioral, systemic, collaborative, narrative, solution-centered, emotion-centered, eco-structural, functional, integrative, and psycho-educational.

In organizing the tasks and work tools, we followed the “4-step family evaluation model”, according to Minuchin [29]. During the 12 meetings, some “steps” were repeated, depending on the identified and/or expressed need.

Techniques and tools were flexibly included, depending on acceptance, openness, newly identified goals, and work pace specific to each parent–child dyad (Table 1).

The individual psychotherapy sessions took place bimonthly for 6 months (12 sessions/50 min each), following the same approach and the same program as in the case of family sessions; with the difference being that only the child/adolescent participated. When debating the problem and identifying the points of view, the connections were made individually by the child/adolescent with the therapist’s support, in the parent’s absence.

### 2.4. Statistical Methods

Statistical analysis was performed using SPSS version 17 (SPSS Inc., Chicago, IL, USA). To compare the difference in the values of the answers to the Parent–Child Relationship Scale between the intervention groups and the control group of parents of children/adolescents with T1DM, we calculated sums, averages, standard deviations, and confidence intervals using the paired-samples *t*-test.

Kolmogorov–Smirnov and Shapiro–Wilk tests were conducted to test the normality of variable distribution. Non-parametric Wilcoxon test for related samples was used to indicate that, at the end of family therapy, significant changes in the mean ranks for closeness and conflict.

## 3. Results

### Descriptive Statistics

In Group 1, FT—Family Therapy, children with T1DM who participated in family systemic therapy sessions together with their parent (caregiver) (N = 22), 45.5% were girls (N = 10) and 54.5% were boys (N = 12). Most of the parents were women (86.4%) (N = 19), men (13.6%) (N = 3), married (68.2%) (N = 15) (C), divorced (9.1%) (N = 2) (D), in a relationship (18.2%) (N = 4) (R), and other civil situations (unmarried, widowed, others) (4.5%) (N = 1). Their mean age was 39.22 years (SD = 6.40). The majority had completed high school education (50%), medium (13.6%), and higher (36.4%), and regarding income, 68.2% declared an average income (N = 15), 4.5% low (N = 1), and 27.3% high (N = 6). In all, 50% (N = 11) of the families lived in urban areas (U) and 50% (N = 11) in rural areas (R) (Table 2).

In Group 2, IT—Individual Therapy, children and adolescents with T1DM who participated in individual psychotherapy sessions (N = 22), 36.4% were girls (N = 8) and 63.6% were boys (N = 14). Most of the parents were women (86.4%) (N = 19), men (13.6%) (N = 3), married (50%) (C) (N = 11), divorced (36.4%) (D) (N = 8), in a relationship (9.1%) (R) (N = 2), and other civil situations (4.5%) (N = 1) (unmarried, widowed, others). Their mean age was 41.18 years (SD = 6.27). The majority had completed high school education (50%) (N = 11), medium (22.7%) (N = 5), and higher (27.3%) (N = 6), and regarding income, those declaring an average income (86.4%) (N = 19), low 4.5% (N = 1), and high 9.1% (N = 2). 40.9% (N = 9) of the families lived in the urban environment (U), and 59.1% (N = 13) in the rural environment (R) (Table 2).

In Group 3, CG—control group, children and adolescents with T1DM who did not participate in any form of therapeutic intervention (N = 20), 25% were girls (N = 5), and 75% were boys (N = 15). Most parents were women (95%), (N = 19), 5% men (N = 1), married (C) (50%) (N = 10), 30% divorced (N = 6) (D), 20% in a relationship (N = 4) (R). They had a mean age of 39.9 years (SD = 6.67). The majority had completed high school education (70%) (N = 14), 20% secondary education (N = 4) and 10% higher education (N-2), and regarding income, 85% declared an average income (N = 17), 10% low (N = 2), and 5% high (N = 1). In all, 40% (N = 8) of the families lived in urban areas (U) and 60% (N = 12) in rural areas (R) (Table 2).

After comparing the average ages of the children in the three groups, we found no differences, the average being 12.09 years (SD = 3.67) in the first group (FT), 12.68 years (SD = 3.19) in the individual psychotherapy sessions (IT), and the mean age of 11.85 years (SD = 4.12 years) in the control group (CG) (Table 3).

The average duration of the T1DM disease was 53.72 months (SD = 24.95) in children from the first group (FT), compared to 76.77 months (SD = 37.17) in the second group (IT), and 53.55 months (SD = 37.92 months) in children from the control group (CG) (Table 3).

Comparison of the HbA1c levels revealed the following results: the first group (FT) had an average value of 8.56% (SD = 1.28), compared to 8.41% (SD = 1.64) in the second group (IT), and 8.17% (SD = 1.49) in the control group (CG) (Table 3).

The medians for children’s ages, duration of the disease and HbA1c’s values are presented in Table 4.

We calculated the differences between the closeness scores and conflict scores from two subscales (dependent variables) between the groups: FT group (systemic family therapy), IT group (individual therapy), and CG (control group) without psychotherapeutic intervention, before (Initial) and after 6 months (Final).

From the results, we found that the mean closeness scores in Group 1, FT (n = 22) before the intervention (m = 19, SD = 5.872) and after the intervention (m = 29.18, SD = 3.984) differed significantly (t = −10.302, df = 21, two-tailed *p* < 0.001). The difference was −10.182. The 95% confidence interval for this difference ranged from −12.237 to −8.126 (Table 5).

We can also state that the mean conflict scores in Group 1, FT before the intervention (m = 32.09, SD = 5.814) and after the intervention (m = 19.2, SD = 4.407) differed significantly (t = 10.774, df = 21, two-tailed *p* < 0.001). The difference was 12.864, the confidence interval for this difference ranging from 10.381 to 15.347 (Table 5).

The non-parametric Wilcoxon test for related samples indicated that, at the end of family therapy, there was a significant increase in the mean ranks for closeness in the subgroup of patients who benefited from family therapy. In the case of the conflict sub-dimension, the same test indicated that, at the end of family therapy, there was a significant decrease in the mean ranks in the subgroup of patients who benefited from family therapy (see Table 6).

Mean closeness scores in Group 1, family therapy (FT) increased significantly after the intervention (final), compared to the mean of the initial scores.

Additionally, mean conflict scores in the same group decreased significantly after the intervention (final), compared to the mean of the initial scores (Table 4). After applying the *t*-test for paired samples to compare the means of closeness and conflict scores before and after individual therapy, we obtained the following results.

The non-parametric Wilcoxon test for related samples indicated that, at the end of individual therapy, there was a significant increase in the mean ranks for closeness in the subgroup of patients who benefited from individual therapy. In the case of the conflict sub-dimension, the same test indicated that, at the end of individual therapy, there was a significant decrease in the mean ranks in the subgroup of patients who benefited from individual therapy (see Table 7).

Following application of the *t*-test for paired samples we compared the means of closeness and conflict scores before and after 6 months in the CG (control group) (see Table 8).

The non-parametric Wilcoxon test for related samples indicated that, at the end of the studied period, there was a slight and insignificant decrease in the mean ranks for closeness in the subgroup of patients who did not receive any form of therapy. In the case of the conflict sub-dimension, the same test indicated that, after 6 months, there was a slight and insignificant increase in the average ranks (mean ranks) in the subgroup of patients who did not receive any form of therapy (see Table 9).

The non-parametric Kruskal–Wallis test indicated that, although initially there was no significant difference between HbA1c levels (χ2 = 2.610, df = 2, *p* = 0.271), after the end of the research therapy/period, the differences became significant in favor of family therapy, followed by individual therapy (χ2 = 9.840, df = 2, *p* = 0.007). After a more thorough analysis using the non-parametric Mann–Whitney U test, it emerged that, at the end of the intervention/period under investigation, the HbA1c levels, expressed as mean ranks, were lower in the group that benefited from family therapy compared to individual therapy (19.59 vs. 25.41, U = 178.0, *p* = 0.132) but without being statistically significant. When the same levels were compared between the subgroups of those with family therapy and those without any type of intervention, the differences became statistically significant, showing a considerable reduction in favor of the first subgroup (16.05 vs. 27.50, U = 100.0, *p* = 0.002). When the comparison was made between the subgroup with individual psychotherapy and that without intervention, HbA1c levels were lower in the former but without a statistically significant difference (18.18 vs. 25.15, U = 147.0, *p* = 0.066).

Following the application of the *t*-test for paired samples, to compare the HbA1c values before (Initial) and after 6 months (Final), depending on the type of intervention, namely, systemic family psychotherapy, individual psychotherapy, and control group (without intervention), we obtained the following results.

Following the results, we can state that the average glycated hemoglobin (HbA1c) level in the group of children and adolescents who participated in systemic family therapy (FT), together with the accompanying parent, before the intervention (M = 8.56, SD = 1.283) and after the intervention (M = 7.45, SD = 1.232) differed significantly from a statistical point of view (t = 10.372, df = 21, two-tailed *p* < 0.001). The difference was 1.114. The 95% confidence interval for this difference ranged from 0.890 to 1.337. The HbA1c means decreased significantly following the family therapy program (after 6 months) (Table 10).

The HbA1c average of the group of children who participated in individual therapy (IT) before the intervention (M = 8.41, SD = 1.644) and after the intervention (M = 7.99, SD = 1.500) was not significantly different (t = 2.016, df = 21, two-tailed *p* = 0.057). The difference was 0.423. The 95% confidence interval for this difference ranged from −0.013 to 0.859. The HbA1c means did not decrease significantly from a statistical point of view following the individual therapy program (after 6 months) (Table 10).

The means of the control group (CG) regarding HbA1c before the intervention (M = 8.16, SD = 1.495) and after 6 months (M = 8.91, SD = 1.812) were not significantly different from a statistical point of view (t = −2.905, df = 19, two-tailed *p* = 0.009). The difference was −0.751. The 95% confidence interval for this difference ranged from −1.293 to −0.209. HbA1c means did not change significantly from a statistical point of view after 6 months without psychological intervention (Table 10).

The results indicate the efficiency of systemic family therapy in improving the quality of parent–child/adolescent relationships, which confirms the first hypothesis. Additionally, the HbA1c was significantly lower after 12 sessions of systemic family therapy, which confirms the second hypothesis.

## 4. Discussion

The problems concerning disease management in children and adolescents with T1DM cannot be addressed without considering the context and environment in which they are identified [30]. The influence of family factors has been highlighted in other chronic disease studies, including diabetes, demonstrating a strong relationship between perceived stress, involvement in solving problems, and adherence to treatment or specialist recommendations [15,31,32].

Starting with this premise, the intended purpose of the current study was to determine the role of the systemic family psychotherapeutic approach in increasing the quality of the parent–child/adolescent relationship and in optimizing glycemic control. There are many studies in the literature that focus on the investigation of parent–child collaboration regarding diabetes, the emphasis being more on cognition and behaviors and less on building a fundamental relationship of trust and safety [33,34]. Others concluded that an accurate analysis and effective intervention must take into account the children’s emotions to increase glycemic balance [35]. On the other hand, caregivers must receive the necessary support and tools to manage crisis situations [36].

Systemic family psychotherapy (SFP) creates a framework in which both children and their parents have the opportunity to express their thoughts and emotions, which in time brings them closer and helps them understand and support each other [37]. The causes of negative emotions cannot always be attributed to strained family relationships, but their processing can influence the way these are managed [37]. In our study, this type of therapy (SFP) proved superior to individual intervention and the control group, and it could be a starting point for the medical system in Romania to evaluate and provide the most effective type of psychological intervention.

Additionally, the parents and children mutually established a hierarchy of problems they wanted to solve. This flexibility in addressing issues is beneficial because it responds to the actual needs of each family member. Regarding the management of diabetes in children, the importance of openness, trust, support, and the other positive attitudes included in the closeness subscale is supported by other researches [38,39]. A strong and stable parent–child/adolescent relationship can support them in decision making, choosing friends, developing a healthy attachment style with others, committing to personal development and academic performance, avoidance or risk reduction behaviors (use of psychoactive substances, smoking, and alcohol consumption) [30].

The positive evaluation of the presented results will encourage the specialist to continue this research with a larger sample, using different types of psychological intervention and more dependent variables.

Improving the HbA1c values means paying more attention to the factors that influence it; that is, diet, correct establishment of insulin requirements, correct and frequent measurements of blood glucose, or the use of CGM (Continuous Glucose Monitoring), where possible [1,4]. A significantly decreased HbA1c value after 6 months indicates a considerable improvement in glycemic control and a decrease in the risk of complications in the short, medium, and long terms, as demonstrated by the studies in the field [3].

The decrease in glycosylated hemoglobin by 0.42 points after 6 months can be considered a therapeutic success, due to the health effects [3].

In the control group, the HbA1c value, higher by 0.75 points, indicated a decrease in glycemic control. Even if this result cannot be considered statistically significant, in the management of DM type 1 in children and adolescents this increase in glycated hemoglobin above the recommended threshold of 7% may contribute to the early onset of disease complications such as ketoacidosis and micro- and macrovascular complications [1,3].

The strong positive correlation between the improvement in the parent–child relationship and the decrease in the HbA1c value supports the considerable positive effect of this type of intervention in the management of diabetes in children and adolescents.

The limitation of this study is that, from a statistical point of view, it does not provide comparisons and correlations among several independent factors, such as demographic factors, age, and gender. This is due to the relatively small number of participants.

Following the results of this study, we propose to continue and improve this experimental design for a longer period of time with a higher number of participants and the inclusion of other important variables. The present study supports pediatric diabetologists to highlight the role of identifying the barriers related to the parent–child relationship in the management of the disease, and therapeutic success.

## 5. Conclusions

The quality of parent–child relationships plays an important role in the management of special life situations such as chronic diseases. Thus, the management of diabetes depends on personal, social, and family resources and their efficiency. Strengthening this relationship through the support, understanding, and warmth provided by parents to their children is the prerequisite for forming a positive self-care attitude and overcoming the barriers to achieving a glycemic balance. Communicating thoughts and emotions, openness, building trust and self-control are skills with long-term positive effects. Systemic family psychotherapy can play an important role in the management of the disease and in providing trust and self-confidence for children and adolescents with type 1 DM.

## Figures and Tables

**Table 1:** Systemic family psychotherapy program.

Session	Objectives	Techniques/Tools
I	Establishing the therapeutic relationship	The therapeutic framework constructive listening, staging, therapeutic alliance.
II	Exposure of the problem	The bearer of the symptom, the role of the symptom, individual perspectives.
III	Identifying the relations that favor and maintain the problem	Family structure, family life cycle, genogram, family map, subsystems, borders, coalitions, attachment.
IV	Identifying the problem’s connections with the past	Lifeline, genogram, patterns, differentiations, non-differentiations, individual perspectives, the life story, metaphor.
V	Identifying different ways of relating	Reframing, searching for new options, competency training, homework.
VI	Relationships in the family organization	Boundaries, alliances, triangulations, parented child, family drawing, buildings, statue.
VII	Identifying the problem’s connections with the present	Narrative technique, role play. The statue, paradoxical intervention, symptom prescription.
VIII	Restructuring interactions	Reframing, perspective, individual perspectives, previous successes, summarization.
IX	Identifying problem’s current connections	Symptom, borders, differentiations, lifeline.
X	Identifying the relations that favor and maintain the problem	Statue, story, diary.
XI	Identifying different ways of relating	Individual perspectives.
XII	“Family self-therapy”—parental involvement and children collaboration in experiencing therapeutic techniques adapted to different current context and personal style and developing problem-solving skills	Role exchange, symptom prescription, reframing, skills training, self-evaluation, new map of the family, metaphors, story (narrative).

**Table 2:** Demographic data of the children and parents participating in the study.

Total	FT	IT	CG
	Family Therapy Group	Individual Therapy Group (Children)	Control Group—without Intervention
(N = 64)	(N = 22)	(N = 22)	(N = 20)
Girls	10 (45.5%)	8 (36.4%)	5 (25%)
Boys	12 (54.5%)	14 (63.6%)	15 (75%)
Area			
Urban	11 (50%)	9 (40.9%)	8 (40%)
Rural	11 (50%)	13 (59.1%)	12 (60%)
Parent gender			
F	19 (86.4%)	19 (86.4%)	19 (95%)
M	3 (13.6%)	3 (13.6%)	1 (5%)
Parent age			
(Years)	39.22 (6.40)	41.18 (6.26)	39.9 (6.67)
Education:			
Secondary	3 (13.6%)	5 (22.7%)	4 (20%)
High school	11 (50%)	11 (50%)	14 (70%)
Higher education	8 (36.4%)	6 (27.3%)	2 (10%)
Marital status:			
Married	15 (68.2%)	11 (50%)	10 (50%)
Divorced	2 (9.1%)	8 (36.4%)	6 (30%)
In a relationship	4 (18.2%)	2 (9.1%)	4 (20%)
Other situation	1 (4.5%)	1 (4.5%)	0 (0%)
Income:			
Low	1 (4.5%)	1 (4.5%)	2 (10%)
Medium	15 (68.2%)	19 (86.4%)	17 (85%)
High *	6 (27.3%)	2 (9.1%)	1 (5%)

Note: data were described in percentages. * Low net income (RON < 1524); medium net income (RON < 3565); high net income (RON > 3565). RON is the Romanian currency.

**Table 3:** Demographic characteristics of children with DM Type 1 from the three groups.

Total	FT	IT	CG
	Family Therapy Group	Individual Therapy Group (Children)	Control Group—without Intervention
N = 64	N = 22 Average (SD)	N = 22 Average (SD)	N = 20 Average (SD)
Children’s ages (years)	12.09 (3.67)	12.68 (3.19)	11.85 (4.12)
Duration of the disease/T1DM (months)	53.72 (24.95)	76.77 (37.17)	53.55 (37.92)
HbA1c value	8.56 (1.28)	8.41 (1.64)	8.17 (1.49)

Note: data were described as mean, standard deviation.

**Table 4:** Demographic characteristics of children with DM Type 1 from the three groups.

Total	FT	IT	CG
	Family Therapy Group	Individual Therapy Group (Children)	Control Group —without Intervention
N = 64	N = 22	N = 22	N = 20
Children’s ages (years)	12.50 (9–15.25)	13.00 (9–15.25)	11.50 (8–15.75)
Duration of the disease/T1DM (months)	53.50 (28.25–72.50)	72.00 (53.75–101.75)	45 (25.25–74.75)
HbA1c value	8.00 (7.77–9.35)	7.95 (7.45–8.85)	7.65 (7.12–9.30)

Note: data were described as median, lower and upper quartiles.

**Table 5:** Differences regarding the parent–child relationship after systemic family psychotherapeutic intervention (FT).

Variable	M/SD Initial	M/SD Final	Confidence Interval 95%		t	df	Sig.	d
			Lower Limit	Upper Limit				
Closeness	19.00 ± 5.872	29.18 ± 3.984	−12.237	−8.126	−10.302	21	0.001	2.195
Conflict	32.09 ± 5.814	19.23 ± 4.407	10.381	15.347	10.774	21	0.001	2.296

Note: data were described as mean, standard deviation, confidence interval, t-value, degree of freedom, significance and effect size; statistical significance exists for values < 0.001 (sig2-tailed).

**Table 6:** Differences in the parent–child relationship after systemic family psychotherapeutic intervention (FT).

Variable	Median Initial (Lower and Upper Quartiles)	Median Final (Lower and Upper Quartiles)	z	Sig.	N
Closeness	16.50 (13.50–21.25)	30.50 (26.75–32.00)	−4.114	<0.001	22
Conflict	34.00 (28.75–37.00)	17.50 (16.00–23.00)	−4.112	<0.001	22

Note: Wilcoxon signed-rank test.

**Table 7:** Differences in the parent–child relationship after individual psychotherapeutic intervention (IT).

Variable	Median Initial (Lower and Upper Quartiles)	Median Final (Lower and Upper Quartiles)	z	Sig.	N
Closeness	19.50 (14.00–24.00)	26.00 (19.75–29.00)	−3.955	<0.001	22
Conflict	26.00 (17.75–34.25)	20.50 (16.00–26.75)	−3.098	0.002	22

Note: Wilcoxon signed-rank test.

**Table 8:** Differences in the parent–child relationship after 6 months without intervention (CG).

Variable	M/SD Initial	M/SD Final	Confidence Interval 95%		T	df	Sig.	d
			Lower Limit	Upper Limit				
Closeness	80.75 ± 15.874	21.15 ± 8.022	53.291	65.909	19.772	19	0.001	4.421
Conflict	28.50 ± 7.776	29.60 ± 7.007	−3.079	0.879	−1.163	19	0.259	0.260

Note: data were described as mean, standard deviation, confidence interval, t-value, degree of freedom, significance, and effect size; statistical significance exists for values <0.001 (sig2-tailed).

**Table 9:** Differences in the parent–child relationship after 6 months—without intervention (CG).

Variable	Median Initial (Lower and Upper Quartiles)	Median Final (Lower and Upper Quartiles)	z	Sig.	N
Closeness	21.50 (12.25–24.75)	19.50 (14.75–29.00)	−2.519	0.012	20
Conflict	30.50 (22.00–34.50)	31.50 (25.50–34.75)	−0.629	0.529	20

Note: Wilcoxon signed-rank test.

**Table 10:** Comparisons of initial and final HbA1c values by intervention type.

Intervention Type	M/SD HbA1c Initial	M/SD HbA1c Final	Confidence Interval 95%		t	df	Sig.	d
			Lower Limit	Upper Limit				
Systemic family psychotherapy	8.56 ± 1.283	7.45 ± 1.232	0.890	1.337	10.372	21	0.000	2.20
Individual psychotherapy	8.41 ± 1.644	7.99 ± 1.500	−0.013	0.859	2.016	21	0.057	0.42
Control group (without intervention)	8.165 ± 1.495	8.916 ± 1.812	−1.293	−0.209	−2.905	19	0.009	0.64

Note: data were described as mean, standard deviation, confidence interval, t-value, degree of freedom, significance and effect size; statistical significance for values < 0.001 (sig 2-tailed).

## Data Availability

Data are unavailable due to privacy or ethical restrictions.
